# Mesoporous Silica Promotes Osteogenesis of Human Adipose-Derived Stem Cells Identified by a High-Throughput Microfluidic Chip Assay

**DOI:** 10.3390/pharmaceutics14122730

**Published:** 2022-12-06

**Authors:** Xin Chen, Chao Wang, Min Hao, Hang Zhao, He Xia, Liyang Yu, Dong Li, Jichuan Qiu, Haijun Li, Lin Han, Yuanhua Sang

**Affiliations:** 1 State Key Laboratory of Crystal Materials, Shandong University, Jinan 250100, China; 2 Institute of Marine Science and Technology, Shandong University, Tsingdao 266237, China; 3 Cryomedicine Laboratory, Qilu Hospital of Shandong University, Jinan 250012, China; 4 Key Laboratory of Cardiovascular Proteomics of Shandong Province, Department of Geriatric Medicine, Qilu Hospital, Shandong University, Jinan 250012, China

**Keywords:** osteogenic differentiation, human adipose-derived stem cells, mesoporous silica, microfluidic detection

## Abstract

Silicon-derived biomaterials are conducive to regulating the fate of osteo-related stem cells, while their effects on the osteogenic differentiation of human adipose-derived stem cells (hADSCs) remain inconclusive. Mesoporous silica (mSiO_2_) is synthesized in a facile route that exhibited the capability of promoting osteogenic differentiation of hADSCs. The metabolism of SiO_2_ in cells is proposed according to the colocalization fluorescence analysis between lysosomes and nanoparticles. The released silicon elements promote osteogenic differentiation. The detection of secretory proteins through numerous parallel experiments performed via a microfluidic chip confirms the positive effect of SiO_2_ on the osteogenic differentiation of hADSCs. Moreover, constructed with superparamagnetic iron oxide (Fe_3_O_4_), the magnetic nanoparticles (MNPs) of Fe_3_O_4_@mSiO_2_ endow the cells with magnetic resonance imaging (MRI) properties. The MNP-regulated osteogenic differentiation of autologous adipose-derived stem cells provides considerable clinical application prospects for stem cell therapy of bone tissue repair with an effective reduction in immune rejection.

## 1. Introduction

Osseous tissue constitutes the skeletal system, which protects and supports the normal life activities of an organism [1]. Several bone tissue defects caused by infection, trauma, tumors, and other bone diseases usually become challenging due to the limited self-repairing ability [1,2]. Typically, stem cells could act as the seed cell sources of tissue engineering owing to their self-renewal and differentiation ability; thus, achieving the fate determination of stem cells plays a crucial role in tissue repair [3].

Mesenchymal stem cells (MSCs), multipotent stem cells, can undergo multidirectional differentiation into mature cell types such as osteoblasts, chondrocytes, and adipocytes [4]. MSCs can be isolated and prepared from bone marrow [5], fat [6], synovial membrane [7], muscle [8], and other tissues [9]. Currently, bone marrow-derived mesenchymal stem cells (BMSCs) are the most widely used and are considered an effective resource for cell-based therapeutics of bone tissue repair [10]. However, BMSCs are difficult to obtain and damaging to patients, and it is impossible to extract enough bone marrow even from healthy donors. Moreover, transplantation to allogeneic individuals may cause an immune response. These issues restrict the clinical application of BMSCs [11]. As a type of MSCs, human adipose-derived stem cells (hADSCs) isolated from adult adipose tissue are easily accessible. Their advantage of autologous sources can significantly reduce the immunoreaction of cell transplantation [12]. Therefore, the osteogenic differentiation and bone repair of hADSCs have attracted extensive attention, especially for their regulation with biomaterials [13]. For example, the hydroxyapatite/collagen hybrid scaffold exhibited a high osteoinductivity effect in hADSCs by promoting matrix mineralization and the expression of the osteo-specific markers alkaline phosphatase (ALP) and osteocalcin (OCN) [14]. After treatment with the same recombinant human bone morphogenetic protein-2 (rhBMP-2) protein, the multiwalled carbon nanotubes were reported to better promote the osteogenic differentiation of hADSCs compared with nanohydroxyapatite both in vivo and in vitro, which was attributed to their activation of Notch-involved signaling pathways and stimulation of hADSCs to form inductive bone by concentrating more specific bone-inducing proteins [15]. Additionally, nanomaterials composed of inorganic elements such as silicon (Si) [16,17], calcium (Ca) [18,19], zinc (Zn) [20], magnesium (Mg) [21], etc., have been increasingly studied in bone tissue repair. Specifically, Si is an essential trace element supporting the normal growth of bone, cartilage, and connective tissue [22]. Due to its decent biocompatibility and osteogenesis efficiency, Si has previously been incorporated into various biomaterials and is regarded as an effective candidate for bone regeneration [23,24]. For example, silk fibroin nanofibers containing silica nanoparticles have been fabricated to promote MC3T3-E1 cell osteogenic differentiation and treat bone defects [25]. Nanocomposites prepared by silica-coated graphene oxide nanosheets have augmented the osteogenic capability of human MSCs via the enhancement of the BMP-SMAD1/5 signaling pathway [26].

In recent years, mesoporous silica nanoparticles (mSiO_2_, MSNs) have attracted much attention in the biomedical field due to their excellent biocompatibility, large specific surface areas, and tunable pores for outstanding drug loading ability [27,28]. The mesoporous structure of SiO_2_ would benefit from its gradual release of Si and thus might be a good candidate to regulate the osteogenic differentiation of hADSCs. Moreover, iron oxide nanoparticles (Fe_3_O_4_ NPs) with superparamagnetism are good T_2_ contrast agents in nuclear magnetic resonance (NMR). The mesoporous structure coating improves the dispersibility in aqueous solution and protects the Fe_3_O_4_ cores from degradation, thus giving the biomaterial and the treated cells NMR properties [29].

The effect of material-derived cell fate regulation is always discussed according to well-established cell analysis methods at the gene and protein levels, such as real-time quantitative polymerase chain reaction (RT–qPCR), immunofluorescence staining, and Western blotting [30]. These experiments provide a solid result for cell assays at the level of large cell populations, which can give the general deduction of cell fate regulation. However, it is almost impossible to realize thousands of parallel experiments at the same time. The limited parallel experiments are likely the reason for the inconclusive results. Therefore, advanced platforms that enable the combination of cell culture and cell assays, performing massively parallel experiments, have attracted increasing attention in recent years [31].

Herein, magnetic core–shell nanoparticles of a magnetic iron oxide (Fe_3_O_4_) core encapsulated by a mSiO_2_ shell (Fe_3_O_4_@mSiO_2_, MNPs) were developed. Then, osteogenic differentiation in hADSCs was enhanced with MNPs-conditioned culture media. The expression of osteo-specific genes and proteins validated the pro-osteogenic effect of MNPs on hADSCs. The NMR imaging property of MNPs in vitro was studied. Moreover, to confirm the promoted osteogenic differentiation effect of MNPs on hADSCs, a microfluidic chip with thousands of chambers was used to spontaneously perform numerous parallel experiments. The secretion of osteocalcin (OCN) from cells was detected and statistically analyzed to further evaluate the osteogenic property of MNPs in hADSCs.

## 2. Materials and Methods

### 2.1. Materials and Regents

For material synthesis, FeCl_3_·6H_2_O (AR), sodium oleate, n-hexane, oleate acid, 1-octadecene, cetyltrimethylammonium bromide (CTAB), and chloroform were purchased from Macklin (Shanghai, China). Ethanol, sodium hydroxide (NaOH), tetraethyl orthosilicate (TEOS), sodium nitrate (NaNO_3_), and ammonium chloride (NH_4_Cl) were purchased from Sinopharm (Shanghai, China).

For cell experiments, α-minimum essential medium (α-MEM) and penicillin/streptomycin were purchased from Gibco (Carlsbad, CA, USA); fetal bovine serum (FBS) was purchased from BI (Beit-Haemek, Israel); and human basic fibroblast growth factor (bFGF) was purchased from PeproTech (Cranbury, NJ, USA). Calcein acetoxymethyl ester (Calcein AM), propidium iodide (PI), and TRIzol reagent were purchased from Solarbio (Beijing, China). Cell Counting Kit-8 was purchased from Dojindo (Mashiki, Japan). β-Actin, RUNX2, OPN, OCN, and BMP-2 primers for RT–qPCR were purchased from Biosune (Shanghai, China). A reverse transcription kit and an SYBR Green Premix Pro Taq HS qPCR Kit II were purchased from Accurate Biotechnology (Hunan) Co., Ltd. (Changsha, China) OPN (rabbit polyAb 22952-1-AP) and OCN (rabbit polyAb 23418-1-AP) primary antibodies (Goat Anti-Rabbit IgG, ab150077) were purchased from Proteintech (Chicago, IL, USA), and secondary antibodies were purchased from Abcam (Cambridge, UK). 4′,6-Diamidino-2-phenylindole (DAPI), Hoechst, and TRITC phalloidin were purchased from Solarbio (Beijing, China). The LysoTracker Red, Alizarin Red S Staining Kit, BCIP/NBT Alkaline Phosphatase Color Development Kit, and Alkaline Phosphatase Assay Kit were purchased from Beyotime (Shanghai, China). The Human Osteocalcin DuoSet ELISA kit (DY1419-05) was purchased from R&D Systems (Minneapolis, MN, USA).

### 2.2. Synthesis of Fe_3_O_4_ Nanoparticles

Fe_3_O_4_ nanoparticles were synthesized according to a previous synthetic procedure [32,33]. Iron chloride (FeCl_3_·6H_2_O) and sodium oleate were dissolved in a solvent consisting of ethanol, distilled water, and hexane, heated to 70 °C and kept for 4 h. Then, the lower aqueous phase was discarded, and the upper products were washed with distilled water in a separatory funnel. Finally, an iron oleate complex was obtained after evaporating off the hexane.

The iron oleate complex was synthesized as described above, and oleate acid was dissolved in the solvent 1-octadecene. Then, the reaction system was heated to 320 °C and kept for 30 min. The reaction solution was then rapidly cooled to room temperature, and Fe_3_O_4_ nanoparticles were precipitated by adding ethanol. The Fe_3_O_4_ nanoparticles were washed several times and centrifuged with ethanol. Finally, the bottom material was dried, and magnetite powder was obtained.

### 2.3. Synthesis of Fe_3_O_4_@mSiO_2_ Nanoparticles

Four grams of CTAB was dissolved in chloroform and 200 mL of distilled water, and then 200 mg of dried Fe_3_O_4_ nanoparticles was dispersed in the above solution. Then, the chloroform solvent was removed from the mixture by sonication, and 1 mg/mL CTAB-stabilized Fe_3_O_4_ aqueous solution was obtained. Fe_3_O_4_-CTAB aqueous solution was added to the mixed solvents of distilled water and NaOH (2 M), heated to 70 °C, and stirred for 30 min. TEOS was slowly added dropwise into the reaction system and kept for 1.5 h. The synthesized nanoparticles were then centrifuged and washed with distilled water and ethanol several times. To remove the CTAB surfactants from mesopores, the as-synthesized nanoparticles were dispersed in a 95% aqueous ethanol solution containing NaNO_3_:NH_4_Cl with a molar ratio of 1:1, and the mixture was heated at 60 °C for 6 h. The materials were then centrifuged and washed with ethanol, and Fe_3_O_4_@mSiO_2_ nanoparticles were obtained.

For material characterization, the morphology of the Fe_3_O_4_@mSiO_2_ nanoparticles was observed by SEM (Hitachi, S-4800, Tokyo, Japan) and TEM (JEM-2100, Tokyo, Japan). The morphology of the Fe_3_O_4_ nanoparticles was observed by Thermo Fisher (Talos F200X, Waltham, MA, USA). XRD patterns were obtained on a Bruker D8 Advance Powder Diffractometer equipped with a Cu Kα sealed tube. FTIR spectra were recorded with a Fourier transform infrared spectrometer (Bruker, Tensor II, Billerica, MA, USA). Magnetization curves were recorded using a vibrating sample magnetometer (LakeShore, 7404, Ouachita Parish, LO, USA).

### 2.4. Cell Culture and Differentiation

In this study, the use of hADSCs was approved by the Research Ethics Committee of Qilu Hospital of Shandong University (Project No. KYLL-2019(KS)-086). hADSCs were extracted from the abdomen adipose tissue of liposuction surgery after obtaining informed written consent from all patients. Specifically, adipose tissue samples were obtained mainly from the subcutaneous fat layer of the abdomen or thighs of an individual healthy female approximately 30 years old. For cell proliferation, hADSCs were cultured in α-MEM supplemented with 10% FBS, 5 ng/mL human bFGF, and 1% penicillin/streptomycin. When being passaged to passages 3–6, cells were used for the following experiments. For cell differentiation, osteogenesis-inducing supplements with 100 × 10^−9^ M dexamethasone, 10 × 10^−3^ M β-glycerophosphate and 50 × 10^−6^ M L-ascorbic acid were added to the basic growth medium without human bFGF. Meanwhile, different concentration of MNPs was added to the culture medium and incubated with hADSCs. hADSCs were maintained in a humidified incubator with 5% CO_2_ at 37 °C, and the culture medium was changed every 2 days.

### 2.5. Live/dead Cellular Staining

After hADSCs were seeded at an initial density of 1 × 10^4^ cells/well and cultured with MNPs for 3 days in 24-well plates, the medium was replaced with 600 μL α-MEM plus 0.5 × 10^−6^ M calcein AM and 3 × 10^−6^ M PI. After incubation at 37 °C for 15 min, the cells were washed three times with phosphate-buffered solution (PBS) and observed with an inverted fluorescence microscope (Olympus, Japan).

### 2.6. CCK-8 Assay

After hADSCs were seeded at an initial density of 3 × 10^3^ cells/well and cultured with MNPs for 1, 2, and 3 days in 96-well plates, the medium was replaced with 100 μL of α-MEM, and 10 μL of CCK-8 solution was added. After the cells were incubated for 1 h at 37 °C, the supernatant was transferred to another 96-well plate, and the optical density value at a wavelength of 450 nm was detected by a CMax Plus microplate reader.

### 2.7. Cytoskeleton Staining

hADSCs were seeded at an initial density of 1 ×10^4^ cells/well and cultured with MNPs for 3 days in 24-well plates. The cells were washed three times with PBS, fixed with 4% paraformaldehyde solution for 10 min, and permeabilized with 0.1% Triton X-100 for 5 min. Then, the cells were blocked with 1% bovine serum albumin (BSA) solution for 1 h at room temperature, followed by culture with TRITC phalloidin at a 1:200 dilution for 30 min to stain the cytoskeleton and DAPI at a 1:1000 dilution for 5 min to stain nuclei. After, the cells were washed with PBS and observed by CLSM (Leica, TCS SP8, Wetzlar, Germany).

### 2.8. Colocalization of Lysosomes and MNPs

hADSCs were seeded in a 24-well plate at an initial density of 1 × 10^4^ cells/well. After cell adherence, MNPs were added to the wells. Subsequently, the cells were washed with PBS three times, followed by culture with LysoTracker Red at a 1:15,000 dilution for 30 min and Hoechst at a 1:1000 dilution for 5 min. Then, the cells were observed by CLSM (Leica, TCS SP8, Wetzlar, Germany).

### 2.9. RT–qPCR Analysis

After being cultured for 7, 14, and 21 days, the cells were lysed with TRIzol reagent, and total RNA was extracted according to the manufacturer’s instructions. RNA concentration and purity were measured by a Q-5000 spectrophotometer (Quawell, Q-5000, San Jose, CA, USA) at 260/280 nm. After reverse transcription, the mRNA levels of one housekeeping gene, β-actin, and four osteogenesis-related genes, RUNX2, OPN, OCN, and BMP-2 (Table S1 for primer sequence), were measured with a real-time quantitative PCR system (Roche, Light-Cycler 480 II, Switzerland). The relative transcript levels of target gene expression were normalized to that of β-actin and expressed as the mean ± SD (*n* = 3 for each group).

### 2.10. Immunofluorescence Staining

hADSCs were seeded at an initial density of 1 ×10^4^ cells per well and cultured with MNPs for 7 and 14 days in 24-well plates. After washing with PBS three times, the cells were prefixed with 4% paraformaldehyde solution for 10 min at room temperature. Then, the cells were permeabilized with 0.1% Triton X-100 for 5 min, followed by blocking with 1% BSA solution for 1 h at room temperature. After, the cells were incubated with primary antibodies against OPN (rabbit polyAb 22952-1-AP, Proteintech, Chicago, IL, USA) at a 1:200 dilution and OCN at a 1:500 dilution (rabbit polyAb 23418-1-AP, Proteintech, Chicago, IL, USA) overnight at 4 °C. The corresponding goat anti-rabbit secondary antibody (ab150077, Abcam, Cambridge, UK) at a 1:1000 dilution was added to stain OPN and OCN for 1 h at room temperature. After being washed three times with PBS, the cells were stained with TRITC phalloidin at a 1:200 dilution for 30 min and DAPI at a 1:1000 dilution for 5 min. Finally, confocal laser scanning microscopy was used to capture fluorescence images.

### 2.11. Identifications of Alkaline Phosphatase (ALP)

ALP expressed from osteoblasts could be detected by an ALP color development kit (Beyotime, Shanghai, China) and ALP assay kit (Beyotime, Shanghai, China) after hADSCs were cultured with MNPs for 7 and 14 days.

### 2.12. Alizarin Red S Staining

After hADSCs were cultured with MNPs for 14 days, the mineralized nodules of osteoblasts were stained with an alizarin red S staining kit (Beyotime, Shanghai, China) according to the manufacturer’s instructions.

### 2.13. Fabrication of PDMS Chips

The mold to fabricate PDMS chips was generated on a silicon wafer that had been etched according to a designed pattern. The surface of the silicon wafer surface was treated with trimethylchlorosilane to facilitate the peel-off of PDMS. Then, the PDMS precursor was completely mixed with the curing agent at a ratio of 10:1, followed by removal of the bubbles in a vacuum dryer for 1 h. Moreover, the silicon plate was clamped with the acrylic plate mold, and the PDMS mixture was injected into the silicon plate mold with a syringe. Finally, PDMS chips were developed after heating at 80 °C for 1 h.

### 2.14. The Array of Antibody Barcodes

For the antibody barcode array, a PDMS chip designed for antibody barcode printing was assembled onto a functionalized glass slide. Two microliters of human osteocalcin capture antibody (R&D system, America) was injected into the microchannels on one side, followed by aspiration from the other side of the microchannels by a vacuum pump, allowing the antibody solution to flow through the microchannels and finally achieve uniform distribution of the capture antibody. After the capture antibody was immobilized on the glass substrate, the PDMS chip was removed, and the substrate was blocked with 3% BSA solution for 10 min to reduce nonspecific adsorption. Then, the functionalized glass slide with the antibody barcode was scanned at an excitation wavelength of 488 nm using Genepix 4400A scanners (Molecular Devices, San Jose, CA, USA) to verify the integrity and accuracy of the antibody barcode.

### 2.15. Secretory Protein Detection

After hydrophilic treatment on PDMS chips for cell culture, hADSCs were seeded at a density of 2 × 10^4^ cells per PDMS chip. Then, the PDMS chips were placed in cell culture dishes, immersed in cell culture medium and kept in a humidified incubator with 5% CO_2_ at 37 °C to maintain normal cell viability. On Day 5, the PDMS chips were gently sealed with antibody-barcode functionalized glass slides. After another 2 days of culture for protein secretion and capture, the glass substrates were stripped away from the PDMS chips, immediately immersed and flushed with 1% BSA solution. Biotinylated detection antibody was then diluted at a ratio of 1:200 in 1% BSA solution, covered onto the glass substrates and incubated for 45 min at room temperature. After, the substrates were washed with 1% BSA solution and incubated with APC dye-labeled streptavidin diluted in 1% BSA solution for 30 min. Finally, the substrates with detection fluorescence signals were washed with 1% BSA, PBS, and DI water successively, followed by laser scanning and fluorescence intensity analysis at an excitation wavelength of 635 nm using Genepix 4400A scanners (Molecular Devices, San Jose, CA, USA).

### 2.16. Statistical Analysis

Statistical analysis was performed with Origin 2018, GraphPad Prism 8.4 and ImageJ software. The values are presented as the mean ± standard deviation (SD). Data were analyzed by one-way or two-way analysis of variance (ANOVA) in GraphPad Prism.

## 3. Results and Discussion

### 3.1. Characterization of MNPs and Their Cytocompatibility in hADSCs

**Figure 1:**
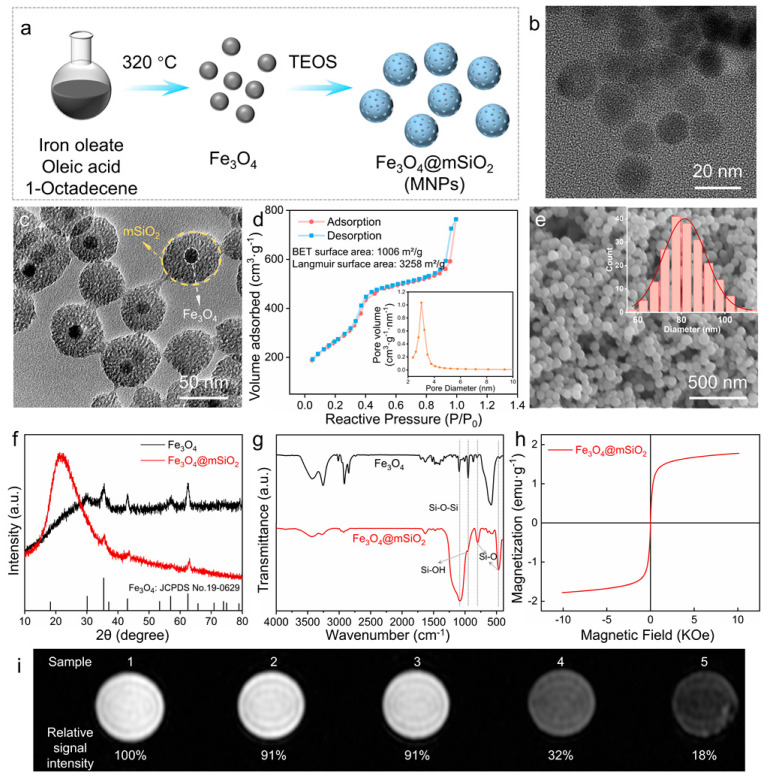
Synthesis and characterization of MNPs. (**a**) Schematic illustration of the preparation process of MNPs. (**b**,**c**) Transmission electron microscopy (TEM) images of Fe_3_O_4_ and MNPs, respectively. (**d**) N_2_ adsorption–desorption isotherms and mesopore size distribution (the inset) of MNPs. (**e**) Scanning electron microscope (SEM) image and particle diameter distribution (the inset) of MNPs. (**f**) The X-ray diffraction (XRD) patterns and (**g**) the Fourier transform infrared spectra (FTIR) images of Fe_3_O_4_ and MNPs. (**h**) Saturation magnetization curve of MNPs at room temperature. (**i**) T_2_-weighted magnetic resonance imaging (MRI) images of different samples. Samples 1 to 5 represent water, silica of 75 and 150 μg/mL, and MNPs of 75 and 150 μg/mL, respectively.

Magnetic nanoparticles (MNPs) with core–shell structures were prepared as illustrated in Figure 1a. Briefly, magnetic iron oxide nanoparticles (Fe_3_O_4_ NPs) were synthesized by the thermal decomposition method of iron oleate complexes and oleic acid in 1-octadecene solutions [32]. Then, tetraethyl orthosilicate (TEOS) was added to the above mixture to synthesize mesoporous silica (mSiO_2_), forming the expected MNPs. The transmission electron microscopy (TEM) image revealed Fe_3_O_4_ NPs ~15 nm in size with good dispersion (Figure 1b). The MNPs showed the core–shell structure formed by the Fe_3_O_4_ NPs and mSiO_2_ (Figure 1c). The encapsulated percentage of MNPs was approximately 70% (Figure S1). The N_2_ adsorption–desorption isotherms belong to type IV curves with a hysteresis loop, demonstrating the mesoporous structure. The BET and Langmuir surface area in the N_2_ adsorption–desorption isotherms (Figure 1d) was calculated to be 1006 m^2^/g and 3258 m^2^/g, respectively. The mesopore size distribution (inset) exhibited a sharp peak around 3 nm. As shown in Figure 1e, the scanning electron microscope (SEM) image confirmed the uniform spherical morphology of MNPs, with an average diameter of 81.48 nm (inset). The X-ray diffraction (XRD) patterns of the obtained Fe_3_O_4_ and Fe_3_O_4_@mSiO_2_ nanoparticles could be assigned to the standard magnetite phase (JCPDS No. 19-0629), and the broad peaks between 10 and 30 degrees were assigned to the amorphous mesoporous silica layer (Figure 1f). The Fourier transform infrared spectra (FTIR) of Fe_3_O_4_ and Fe_3_O_4_@mSiO_2_ nanoparticles are shown in Figure 1g. The absorption peak appearing at ~1080 cm^−1^ was attributed to the antisymmetric stretching vibration of Si-O-Si, the band at approximately 955 cm^−1^ illustrated the bending vibrations of Si-OH, and two sharp peaks at ~800 cm^−1^ and ~470 cm^−1^ were the stretching vibrations of the Si-O bond, indicating the formation of mSiO_2_. The saturation magnetization curves of Fe_3_O_4_ NPs and MNPs showed closed hysteresis loops with the areas approaching zero (Figure S2 and Figure 1h), confirming the superparamagnetic property of the Fe_3_O_4_ NPs and MNPs. The saturation magnetizations of Fe_3_O_4_ NPs and MNPs at room temperature were ~40.6 emu·g^−1^ and ~1.8 emu·g^−1^, respectively. The decrease in the saturation magnetization of MNPs (4.5%) is attributed to the low content of Fe_3_O_4_ NPs in the MNPs, consistent with the 5.0 wt% Fe_3_O_4_ in MNPs during the synthesis. T_2_-weighted MRI images of MNPs and pure silica are shown (Figure 1i and Figure S3). The relative signal intensity of different samples was normalized to that of pure water. Silica at 75 μg/mL, silica at 150 μg/mL, MNPs at 75 μg/mL, and MNPs at 150 μg/mL were ~91%, ~91%, ~32%, and ~18%, respectively. Pure silica had no T_2_-weighted imaging effect, and the decline in signal intensity was due to the T_2_-weighted enhancement of Fe_3_O_4_ NPs in MRI. The superparamagnetic property of MNPs from Fe_3_O_4_ NPs would benefit the NMR imaging property of the modified cells or tissues.

**Figure 2:**
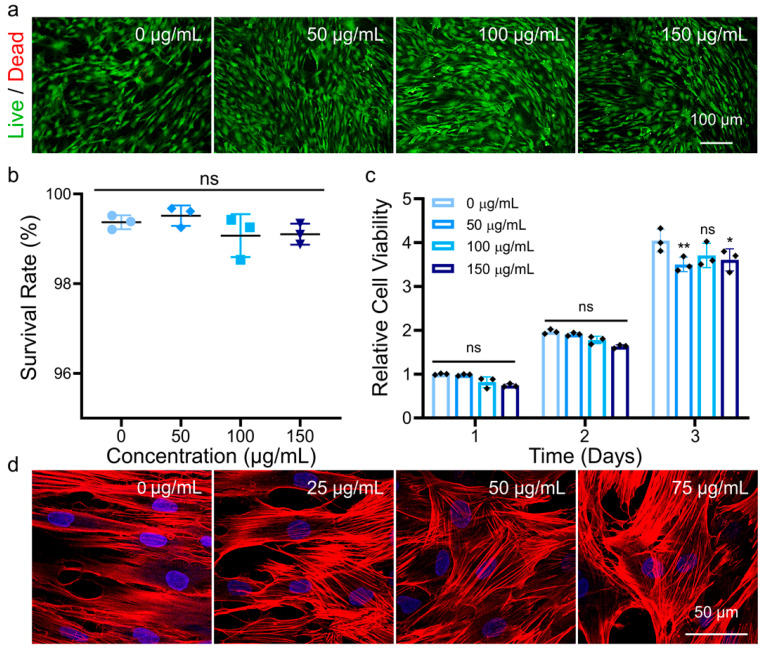
Viability and morphology of hADSCs cultured with MNPs. (**a**) Live/dead cellular staining images of hADSCs after culture with 0, 50, 100, and 150 μg/mL MNPs for 3 days. The live cells were green, and the dead cells were red. (**b**) Survival rate of hADSCs cultured with increasing concentrations of MNPs for 3 days. Data represent mean ± standard deviation (*n* = 3). Significance was determined by one-way ANOVA (ns: not significant vs. the 0 μg/mL group). (**c**) Relative cell viability of hADSCs after incubation with 0, 50, 100, and 150 μg/mL MNPs for 1, 2, and 3 days. Data represent mean ± standard deviation (*n* = 3). Significance was determined by two-way ANOVA (* *p* < 0.05, ** *p* < 0.01, ns: not significant compared with the 0 μg/mL group on Day 1). (**d**) Cytoskeleton staining images of hADSCs cultured with 0, 25, 50, and 75 μg/mL MNPs for 3 days. F-actin was stained red, and nuclei were stained blue.

To evaluate the biological performance of MNPs, we initially studied the cytocompatibility via cell viability and morphology assays, as shown in Figure 2. Live/dead staining and cell counting kit-8 (CCK-8) experiments were used to qualitatively and quantitatively analyze cell viability. Live/dead staining was performed after cells were cultured with 0, 50, 100, or 150 μg/mL MNPs for 3 days (Figure 2a and Figure S4). Live cells were stained green with calcein acetoxymethyl ester (Calcein AM), while dead cells were stained red with propidium iodide (PI). Numerous living cells and only a few dead cells were observed. The average percentages of living hADSCs were counted by ImageJ software after culturing with 0, 50, 100, or 150 μg/mL MNPs, and the results were 99.4%, 99.5%, 99.1%, and 99.4%, respectively (Figure 2b). There was no obvious difference among all groups, demonstrating the good biocompatibility of MNPs in hADSCs. As illustrated in Figure 2c, CCK-8 assays were used to determine cell viability at 1, 2, and 3 days after incubation with 0, 50, 100, and 150 μg/mL MNPs. When cultured with various amounts of MNPs, the cell viability of hADSCs was similar to each other. With continuous culture, the metabolic activity of hADSCs was upregulated from Day 1 to Days 2 and 3, suggesting that the presence of MNPs does not inhibit cellular viability. A lower dosage of MNPs was selected as 25, 50, and 75 μg/mL below 100 μg/mL to further study the material effect on cell fate regulation. Filamentous actin (F-actin), as one of the major components of the cytoskeleton in cells, is generally considered to be involved in cell morphology maintenance, migration, and endocytosis [34]. TRITC phalloidin-tagged F-actin and DAPI-tagged nuclei were stained after 3 days of culture with various concentrations of MNPs (0, 25, 50, 75 μg/mL). The intact cell morphology and cytoskeleton demonstrated the good biocompatibility of MNPs (Figure 2d and Figure S5). With increasing MNPs supplementation, the cell morphology gradually changed from a long spindle shape to a fusiform shape, and this subtle change in the cytoskeleton may be attributed to the early osteogenic effect of MNPs on cells. To confirm the NMR properties of hADSCs modified with MNPs, we cultured cells with 75 and 150 μg/mL MNPs, and T_2_-weighted MRI images were obtained (Figure S6). The results showed that MNPs could be endocytosed by cells and confer NMR function on cells.

**Figure 3:**
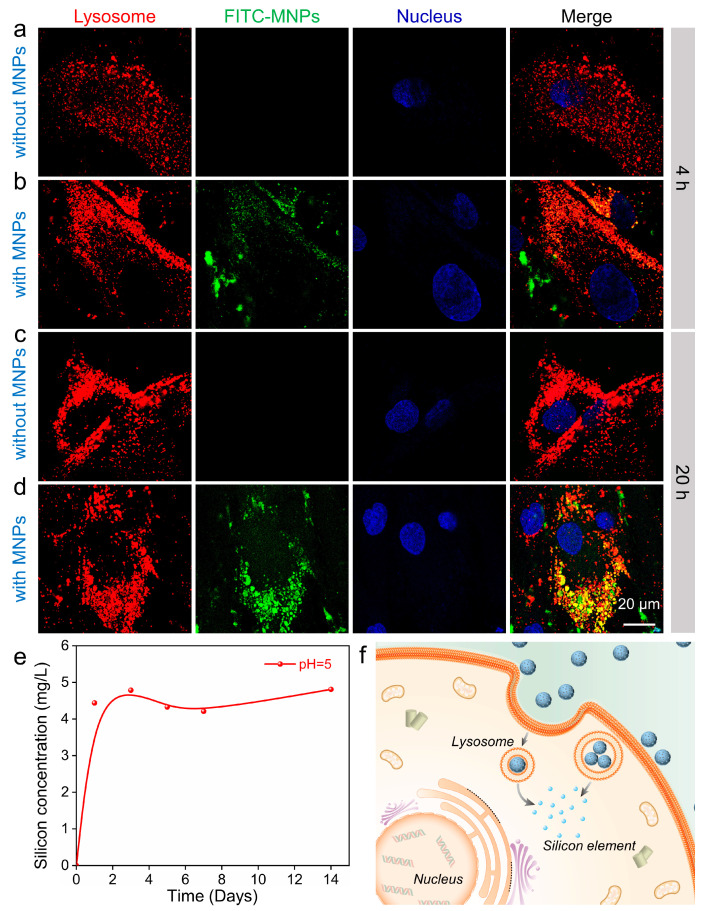
Colocalization between MNPs and lysosomes. Fluorescence images of hADSCs without (**a**) and with MNPs (**b**) for 4 h and cultured without (**c**) and with MNPs (**d**) for 20 h. Lysosomes were stained with LysoTracker (red), MNPs were tagged with FITC (green), and the nucleus was stained with Hoechst (blue). (**e**) Silicon release from MNPs at pH 5 for 14 days. (**f**) Schematic illustration displaying the intracellular processes of MNPs in hADSCs.

Subsequently, the endocytosis of MNPs within hADSCs was visualized. MNPs had an average diameter of ~80 nm, which would be endocytosed via the endosome–lysosome pathway of hADSCs [35]. To validate this hypothesis, the colocalization of lysosomes and FITC-MNPs was examined by confocal laser scanning microscopy (CLSM). The lysosomes were labeled red with LysoTracker, MNPs were stained green after adsorption of fluorescein isothiocyanate (FITC), and the nucleus was tagged blue with Hoechst. In the first 4 h of culture, compared with the group without MNPs (Figure 3a), hADSCs cultured with MNPs showed slight green fluorescence and little colocalization (Figure 3b). This result indicates that hADSCs began the endocytosis of MNPs, but the amount was relatively small. After further culturing until 20 h, compared with the group without MNPs (Figure 3c), most of the FITC-MNPs were located in the areas of lysosomes (Figure 3d). Next, the silicon release resulting from the intracellular metabolism of MNPs needed to be clarified; hence, we performed an in vitro experiment in which MNPs were soaked in a buffer solution at pH 5 for 14 days. The supernatant obtained by centrifugation was collected, and the silicon concentration was measured using an inductively coupled plasma optical emission spectrometer (ICP–OES, Thermo ICAP PRO). Silicon release was fast during the first 2 days and then reached a plateau (Figure 3e). These findings confirmed that the MNPs could be internalized and digested by lysosomes, and the released silicon elements in cells may further affect certain intracellular biological processes (Figure 3f).

### 3.2. Osteogenic Differentiation Regulation of hADSCs with MNPs

**Figure 4:**
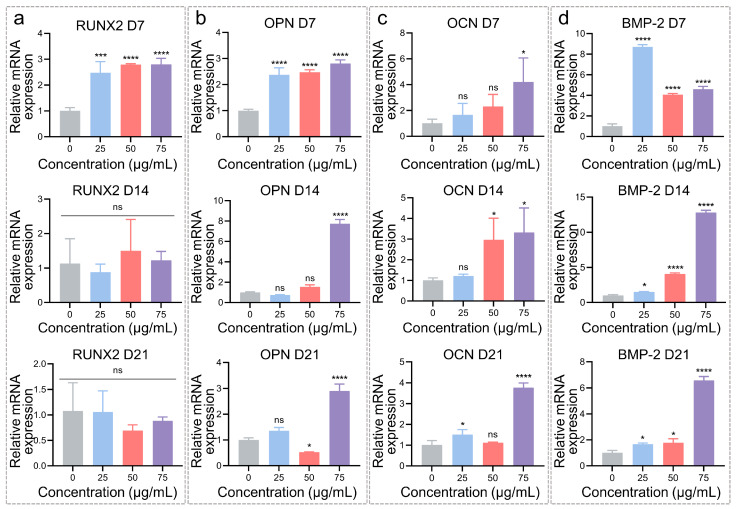
Osteogenic-specific gene expression by real-time quantitative polymerase chain reaction (RT–qPCR) analysis. RUNX2 (**a**), OPN (**b**), OCN (**c**) and BMP-2 (**d**) mRNA expression on Day 7, Day 14, and Day 21. Data represent mean ± standard deviation (*n* = 3). Significance was determined by one-way ANOVA (* *p* < 0.05, *** *p* < 0.001, **** *p* < 0.0001, ns: not significant vs. the 0 μg/mL group).

**Figure 5:**
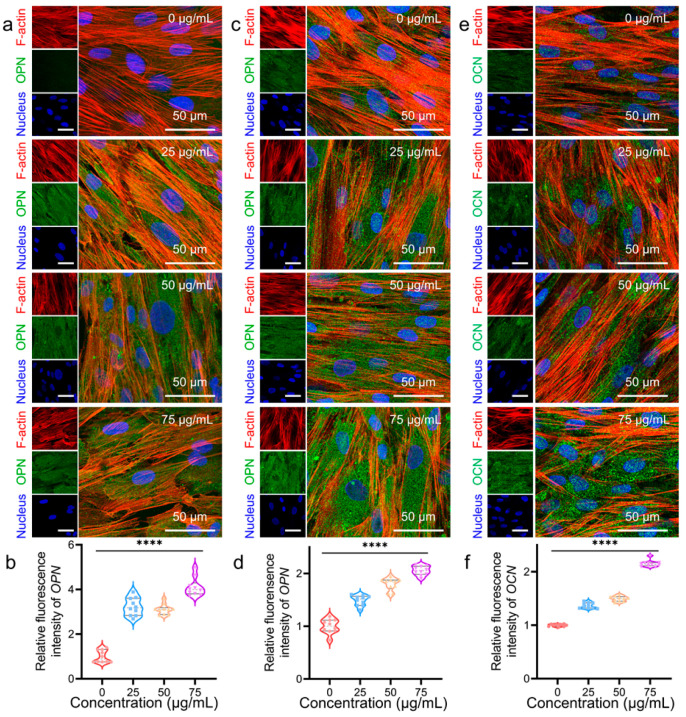
Immunofluorescence staining of OPN and OCN protein. (**a**) OPN (green), F-actin (red), and nuclear (blue) staining images for 7 days. (**b**) Quantitative analysis of the mean fluorescence intensity of immunofluorescence-stained images of OPN expression at 7 days. (**c**) OPN (green), F-actin (red), and nuclear (blue) staining images for 14 days. (**d**) Quantitative analysis of the mean fluorescence intensity of immunofluorescence-stained images of OPN expression at 14 days. (**e**) OCN (green), F-actin (red), and nucleus (blue) staining images for 14 days. (**f**) Quantitative analysis of the mean fluorescence intensity of immunofluorescence-stained images of OCN expression at 14 days. Data represent mean ± standard deviation (*n* = 10). Significance was determined by one-way ANOVA (**** *p* < 0.0001, vs. the 0 μg/mL group).

The defined concentrations (0, 25, 50, and 75 μg/mL) of MNPs were used to regulate the osteogenesis of hADSCs, and the expression of osteo-related genes and proteins was analyzed (Figure 4 and Figure 5). The relative mRNA expression of osteogenic-specific markers was determined by RT–qPCR analysis (Figure 4) after cultivation for 7, 14, and 21 days. As a key transcription factor, runt-related transcription factor 2 (RUNX2) is essential for osteoblast differentiation and drives skeletal development during the early stages [4]. After 7 days of culture with 25, 50, and 75 μg/mL MNPs, RUNX2 expression in hADSCs was 2.5-, 2.8-, and 2.8-fold that in hADSCs cultured with 0 μg/mL MNPs, respectively (Figure 4a). However, on Days 14 and 21, the expression levels of RUNX2 showed little difference, likely attributed to the decreased expression of RUNX2 in mature osteoblasts. Osteopontin (OPN) is another marker expressed during the differentiation of primary osteoblasts. OPN regulates osteoblast adhesion, osteoclast function, and matrix mineralization, ultimately contributing to bone formation and remodeling [36]. As the concentrations of MNPs increased from 25 to 50 and 75 μg/mL, OPN expression was enhanced to 2.4-, 2.5-, and 2.8-fold that of the 0 μg/mL group on Day 7. After 14 and 21 days of culture, the mRNA expression of OPN was upregulated in the 75 μg/mL group and was 7.7- and 2.9-fold that of the 0 μg/mL group, respectively (Figure 4b). This fold change occurs mainly because OPN is an early marker in osteogenesis. Normal cells differentiated into mature osteoblasts after 21 days of culture, so the elevated OPN expression was reduced on Day 21 compared to Day 14. Moreover, it was found that MNPs could exhibit a relatively steady pro-osteogenic function after reaching a certain concentration. OCN is one of the extracellular matrix proteins secreted by cells and generally occurs at late stages of osteogenesis, thus acting as a marker for terminally differentiated osteoblasts [37]. After 7 days of culture, the OCN expression in the 25, 50, and 75 μg/mL groups was 1.7-, 2.3-, and 4.2-fold higher than that in the 0 μg/mL group, respectively. Furthermore, the expression levels of OCN were 1.2-, 3.0-, and 3.3-fold on Day 14 and 1.5-, 1.1-, and 3.8-fold on Day 21, respectively, that of 0 μg/mL (Figure 4c). These results indicate that higher dosages of MNPs were more effective in the regulation of osteoblasts. For osteoblasts, bone morphogenetic protein-2 (BMP-2) maintains osteoblast-specific phenotypes, enhances the upregulation of osteoblast markers, and promotes matrix mineralization [4]. When cultured with different MNPs concentrations, the expression of BMP-2 was obviously enhanced. Especially for 75 μg/mL MNPs, BMP-2 expression was 4.6-fold (Day 7), 12.8-fold (Day 14), and 6.6-fold (Day 21) that of 0 μg/mL, respectively (Figure 4d). Briefly, osteo-related gene expression was generally upregulated with MNP treatment. A low dosage of MNPs (25 μg/mL) initiated the osteogenic differentiation of hADSCs. However, for the further regulation of osteoblasts, a relatively high dosage of MNPs (75 μg/mL) would be needed.

Next, immunofluorescence staining was performed to confirm the protein expression in hADSCs after 7, 14 and 21 days of culture (Figure 5 and Figure S7). OPN and OCN are labeled with green, F-actin is stained red, and the nucleus is stained blue. Meanwhile, quantitative analysis of the mean fluorescence intensity of OPN and OCN was performed, and all groups were normalized to 0 μg/mL. For 7 days, there was higher OPN protein secretion in hADSCs cultured with MNPs than in cells cultured without MNPs (Figure 5a). The mean fluorescence intensity of OPN in 25, 50, and 75 μg/mL MNP-treated hADSCs was ~3.2, ~3.1, and ~4.1-fold higher than that of 0 μg/mL, respectively (Figure 5b). This result is consistent with the gene expression of OPN on Day 7 (Figure 4b). After 14 days, green fluorescence exhibited clear expression among all groups because of the long-term culture. With increasing MNPs concentration, OPN protein expression was still enhanced compared with that without MNPs treatment (Figure 5c). Figure 5d shows the mean fluorescence intensity of OPN after 14 days of culture. OPN protein secretion in 25, 50, and 75 μg/mL MNP-stimulated hADSCs was ~1.5, ~1.9, and ~2.0-fold that of 0 μg/mL, respectively (Figure 5d). After accumulation during the 14-day culture, the secreted OPN proteins showed an increasing trend with the addition of MNPs only. Similar to OPN protein expression, OCN protein expression was enhanced with the MNPs after 14 days of culture (Figure 5e). The mean fluorescence intensity of OCN was ~1.3, ~1.5, and ~2.2-fold that of 0 μg/mL when cultured with 25, 50, and 75 μg/mL MNPs, respectively (Figure 5f). A common tendency was that the mean fluorescence intensity gradually improved with increasing MNPs concentration, which was consistent with the RT–qPCR results.

**Figure 6:**
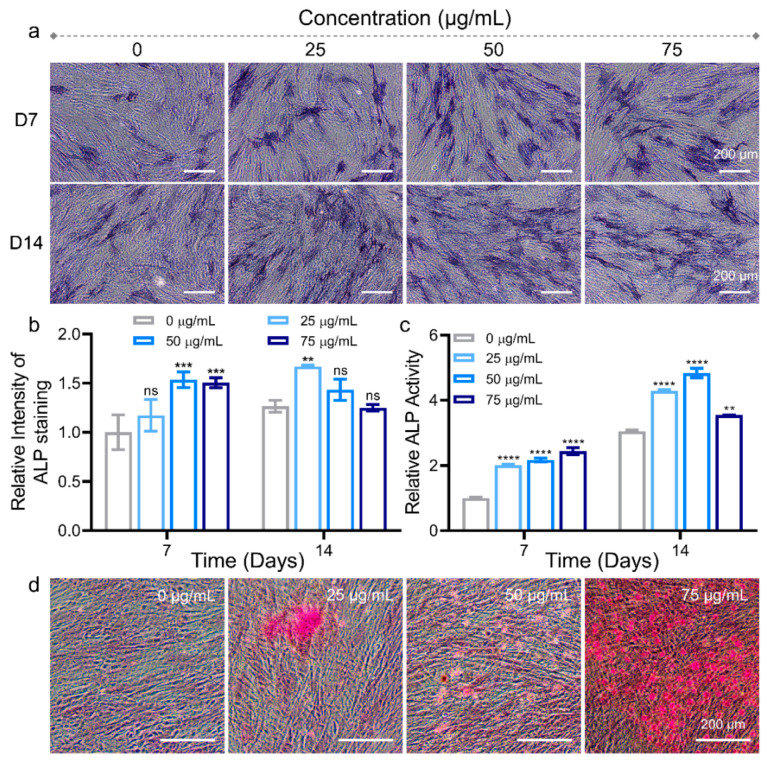
Identification of alkaline phosphatase (ALP) and mineralized nodules in osteoblasts. (**a**) ALP staining images on Days 7 and 14 after being cultured with 0, 25, 50, and 75 μg/mL MNPs. (**b**) Quantitative analysis of ALP staining intensity after being cultured with different concentrations of MNPs. Data represent mean ± standard deviation (*n* = 3). Significance was determined by two-way ANOVA (** *p* < 0.01, *** *p* < 0.001, ns: not significant vs. the 0 μg/mL group at 7 days). (**c**) Relative ALP activity after 7 and 14 days when cultured with 0, 25, 50, and 75 μg/mL MNPs. Data represent mean ± standard deviation (*n* = 3). Significance was determined by two-way ANOVA (** *p* < 0.01, **** *p* < 0.0001, vs. the 0 μg/mL group at 7 days). (**d**) Alizarin Red S staining images of hADSCs cultured with 0, 25, 50, and 75 μg/mL MNPs after 14 days.

Apart from the expression of osteoblast-related genes and proteins, osteogenic differentiation of stem cells can also be identified by detecting the biological markers of osteoblasts (Figure 6). Osteogenesis differentiation at the early stage can be determined by ALP, which acts as an extracellular enzyme for osteoblasts that directly reflects the activity or functional status of osteoblasts [38]. The following stage can be evaluated by analyzing the mineralization of the extracellular matrix [39]. The hydrolysate of 5-bromo-4-chloro-3-indolyl-phosphate (BCIP) and nitro-blue tetrazolium (NBT) was used to perform ALP staining as blue. After culturing with 0, 25, 50, and 75 μg/mL MNPs for 7 days, the color gradually became darker with increasing doses, indicating higher ALP expression at higher doses (Figure 6a). After 14 days of culture, the ALP expression of all groups was comparable. This trend should be attributed to the early stage expression of ALP during osteogenic differentiation. The statistical analysis of ALP staining results is shown in Figure 6b, where all groups were normalized to 0 μg/mL after 7 days of culture. The mean ALP intensity for 25, 50, and 75 μg/mL MNPs after 7 days of culture with hADSCs was 1.2-, 1.5-, and 1.5-fold, while 0, 25, 50, and 75 μg/mL after 14 days of culture with hADSCs was 1.3-, 1.7-, 1.4-, and 1.3-fold, respectively. ALP activity was measured using an ALP assay kit for hADSCs cultured with different dosages of MNPs. Figure 6c shows the relative ALP activity of hADSCs after 7 and 14 days of culture. After 7 days of culture with MNPs, ALP activities in hADSCs of the 25, 50, and 75 μg/mL groups were 2.0-, 2.2-, and 2.4-fold that of the 0 μg/mL group. After 14 days of culture, ALP activities in hADSCs of all groups improved. The values were 3.0-, 4.3-, 4.8-, and 3.6-fold that of the 0 μg/mL group on Day 7, respectively. Notably, the results of ALP staining and activity showed that the expression of ALP was upregulated with the dosages of MNPs at 7 days, while there was a downward trend at higher concentrations of MNPs at 14 days. This phenomenon can be ascribed to the more effective promotion of osteogenesis of higher concentrations of MNPs at an earlier stage, as ALP is an early osteogenic marker and the upregulation of ALP occurs during early osteogenesis [40]. Moreover, it is reported that ALP activity gradually improved during the early stage, then normally declined after cells enter the mineralization stage during osteogenic differentiation in vitro [41].

During osteogenesis, calcium salts developed by calcium ions are deposited on the surface of the cells, forming calcium nodules, also known as bone nodules. Mineralized nodules are signs of osteoblastic differentiation and maturity, as well as the main morphological characteristics of osteoblasts exercising osteogenic function [42,43]. Alizarin red S staining was utilized to analyze mineralized nodules of osteoblasts after 14 days of culture with various dosages of MNPs (Figure 6d). The calcium nodules clearly increased in the hADSCs with the increased dosage of MNPs. The cells cultured with 75 μg/mL MNPs exhibited the most and clearest calcium nodules, indicating an obvious improvement in the osteogenesis of hADSCs.

### 3.3. Microfluidic Detection of MNPs Effects on Osteogenic Differentiation of hADSCs

**Figure 7:**
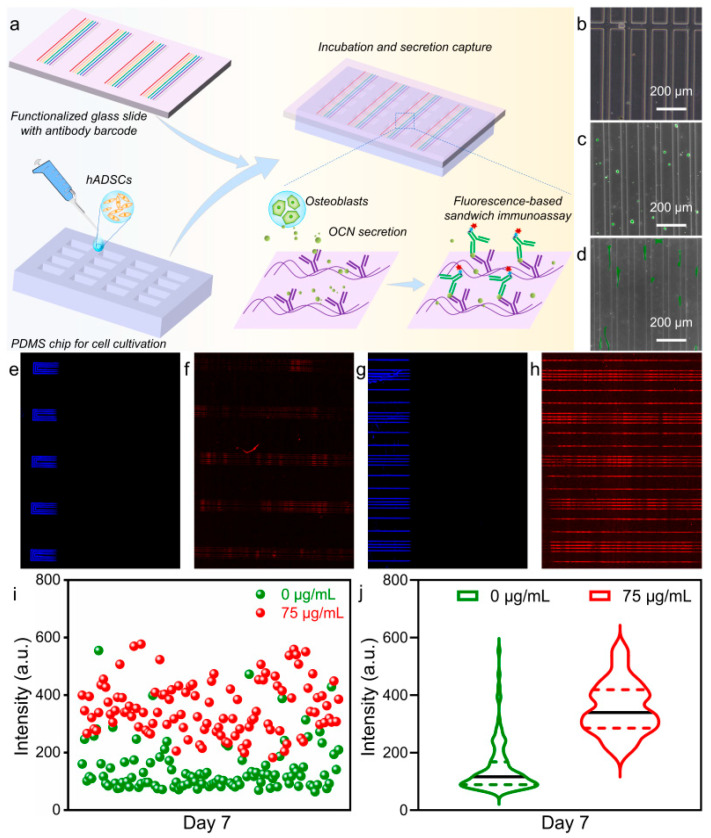
(**a**) Schematic of hADSC-secreted OCN protein detection by a polydimethylsiloxane (PDMS) microfluidic chip and a functionalized glass slide with antibody barcodes. (**b**) Pure PDMS chip in bright field. (**c**) Cell seeded on the PDMS chip at 0 h. (**d**) Cell morphology after being seeded on the PDMS chip for 24 h. Detection of capture antibody spreading on the glass slide (**e**,**g**) and fluorescence signal image of the antibody-barcoded glass slide (**f**,**h**) cultured with 0 and 75 μg/mL MNPs, respectively. (**i**) Scatter diagram showing the distribution of fluorescence intensity after 7 days of cultivation on hADSCs with 0 and 75 μg/mL MNPs. (**j**) Violin plot showing the average fluorescence intensity after 7 days of culture with 0 and 75 μg/mL MNPs on hADSCs.

To further confirm the promoted osteogenic differentiation of hADSCs by MNPs, numerous parallel experiments were performed on a microfluidic chip by detecting the secreted OCN protein of hADSCs (Figure 7). A schematic diagram of the microfluidic chip is shown in Figure 7a. A biocompatible PDMS microchip with more than 7000 microchambers was used as the cell culture microplate. To detect the osteo-specific secretory protein from hADSCs, a glass slide functionalized with antibody barcodes was prepared based on the microfluidic technique. After seeding the hADSC suspension in the microchambers of the PDMS chip, the antibody-modified glass slide was placed onto the PDMS chip. During continued culture, OCN secretion could be captured on the glass slide by a fluorescent sandwich immunoassay based on antigen–antibody-specific binding. A microchamber in a PDMS chip had a cuboid groove structure with a size of 1160 × 100 × 50 μm^3^ (length × width × depth) (Figure 7b). After hydrophilic pretreatment, the seeded cells were well dispersed in the microchambers. There were only several cells located in a chamber, which would reduce the effect of cell–cell interactions, benefiting the study of MNPs-promoted cell fate regulation (Figure 7c). After 24 h of culture, the cells spread well with good adhesion, as shown in Figure 7d. Moreover, cells proliferated and differentiated normally in the PDMS chambers with further culture (Figures S8 and S9).

The antibody-functionalized glass slides were removed from the PDMS chip, and the antibody dispersion was detected on the glass slides. Specifically, fluorescence-labeled IgG antigens were dropped on one end of a glass slide, and the fluorescent signal reading was performed at a wavelength of 488 nm using a Genepix 4400A scanner (Molecular Devices) (Figure 7e,g), which exactly evaluated the homogeneous distribution of the capture antibody. After 5 days of culture of hADSCs with and without MNPs, the antibody-coated slides were placed on PDMS chips and formed enclosed microchambers. The OCN protein secreted in the following 2 days was analyzed. Once OCN protein was combined with the capture antibody, the detection antibody exhibited fluorescence signals that could be read at a wavelength of 635 nm by a Genepix 4400A scanner. The red fluorescent signals illustrated in Figure 7f,h were assigned to the hADSCs cultured with 0 and 75 μg/mL MNPs, respectively. OCN was secreted during the culture of hADSCs with or without MNPs for 7 days. Notably, the detected fluorescent signal was stronger from the hADSCs cultured with 75 μg/mL MNPs. This result was consistent with the above discussion on the promotion of hADSC osteogenesis. To quantitively analyze fluorescent signals, more than 100 areas from fluorescence images were selected randomly. Their fluorescence intensities were read and exported by a Genepix 4400A scanner (Figure S10). The fluorescent intensity distribution of each area is shown in a scatter diagram (Figure 7i). Notably, most fluorescent signal intensities from cells without MNPs were concentrated below 200, while those from cells with MNPs were totally dispersed and spread over 200. Additionally, the average fluorescence intensities are illustrated in violin plots (Figure 7j). The average intensity from cells cultured without MNPs was ~145, while that from those cultured with MNPs was ~355. This value was approximately 2.5-fold that without MNPs. Among these results, we confirmed the promoting property of MNPs on the osteogenesis of hADSCs by detecting OCN secretion. This procedure has precise, high-throughput detection ability and thus can be used as a helpful tool for easily identifying the effective regulation of cell fate with numerous parallel experiments.

## 4. Conclusions

In summary, the synthesized MNPs with silicon elements can promote osteogenesis on hADSCs. An in situ and effective microfluidic chip platform was established for secretory protein detection at the level of numerous parallel experiments, which is a relatively advanced technology in cell analysis potential and detection sensitivity. The colocalization between MNPs and lysosomes implies the silicon metabolism process via endocytosis into the lysosomes of MNPs. Meanwhile, the superparamagnetic Fe_3_O_4_ cores in MNPs confer NMR potential on cells. The regulatory effect of MNPs on the fate of hADSCs was first confirmed using osteo-related gene and protein analysis. Subsequently, microfluidic detection was associated with cell culture, realizing the high-throughput and in situ identification of the degree of cell differentiation. More than 7000 parallel experiments have been performed and provide a reasonable result for the enhanced osteogenic property of MNPs on hADSCs.

## Data Availability

Not applicable.

## Supplementary Materials

The following supporting information can be downloaded at: https://www.mdpi.com/article/10.3390/pharmaceutics14122730/s1, Figure S1: TEM images of MNPs at low magnification; Figure S2: Saturation magnetization curve of Fe_3_O_4_ nanoparticles at room temperature; Figure S3: The original data of T_2_-weighted MRI image of water, silica of 75 and 150 μg/mL and MNPs of 75 and 150 μg/mL; Figure S4: Live/dead cellular staining images after hADSCs were cultured with different concentrations of MNPs for 3 days; Figure S5: Fluorescence images of cell cytoskeleton when hADSCs were cultured with different concentrations of MNPs for 3 days; Figure S6: The original data of T_2_-weighted MRI images of cell and cell cultured with MNPs of 75 and 150 μg/mL; Figure S7: Immunofluorescence staining of OPN and OCN protein after cultured with different concentrations of MNPs 21 days. Figure S8: Immunofluorescence staining of OPN protein after cultured with different concentrations of MNPs 7 days on PDMS chips; Figure S9: Immunofluorescence staining of OCN protein after cultured with different concentrations of MNPs for 14 days on PDMS chips; Figure S10: Illustrations for selecting more than 100 points to analyze fluorescence intensity; Table S1: Sequences of RT-qPCR primers.
