# Supplementary material for: Mesoporous Silica Promotes Osteogenesis of Human Adipose-Derived Stem Cells Identified by a High-Throughput Microfluidic Chip Assay
Source: Pharmaceutics. 2022 Dec 6;14(12):2730. doi: 10.3390/pharmaceutics14122730 (PMC9781822; doi:10.3390/pharmaceutics14122730)
Supplement: Supplementary file 1 [file pharmaceutics-14-02730-s001.zip › pharmaceutics-2031818-supplementary.pdf]

Supplementary Materials

Mesoporous silica promotes osteogenesis of human adipose-derived stem cells identified by a high-throughput microfluidic chip assay

Xin Chen [1], Chao Wang [2], Min Hao [1], Hang Zhao [1], He Xia [1], Liyang Yu [1], Dong Li [3], Jichuan Qiu [1], Haijun Li [4,*], Lin Han [2,*], and Yuanhua Sang [1,*]

[1] State Key Laboratory of Crystal Materials, Shandong University, Jinan, 250100, P. R. China
[2] Institute of Marine Science and Technology, Shandong University, Tsingdao, 266237, P. R. China
[3] Cryomedicine Laboratory, Qilu Hospital of Shandong University, Jinan 250012, P. R. China
[4] Key Laboratory of Cardiovascular Proteomics of Shandong Province, Department of Geriatric Medicine, Qilu Hospital, Shandong University, Jinan, 250012, P. R. China
*Correspondence: navy1629@163.com (H. L.); hanlin@sdu.edu.cn (L. H.); sangyh@sdu.edu.cn (Y. S.)

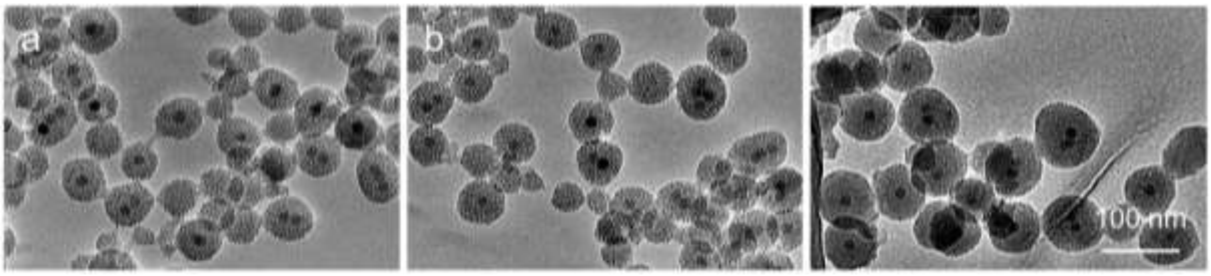

Figure S1. TEM images of MNPs at low magnification.

Three TEM images were selected randomly, the number of MNPs and total nanoparticles was counted, the ratio of MNPs was calculated, and the average was obtained as the final MNPs yield. As shown in Figure S1(a), (b), and (c), the percentage of MNPs was ~71%, 65%, and 74%, respectively, and the average was ~70%.

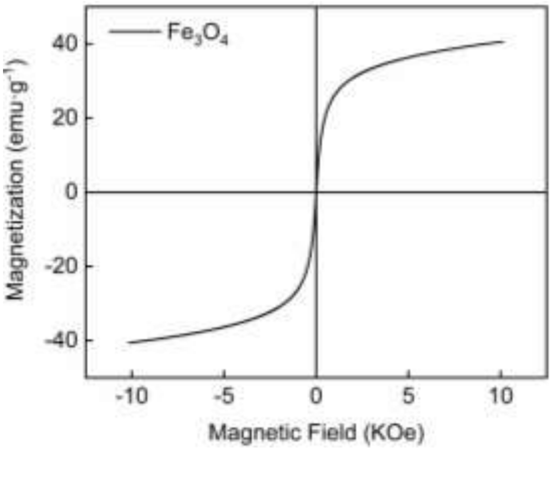

Figure S2. Saturation magnetization curve of Fe_3O_4 nanoparticles at room temperature.

The as-prepared Fe_3O_4 nanoparticles exhibited excellent superparamagnetism with a saturation magnetization of ~40.6 emu·g^{-1}.

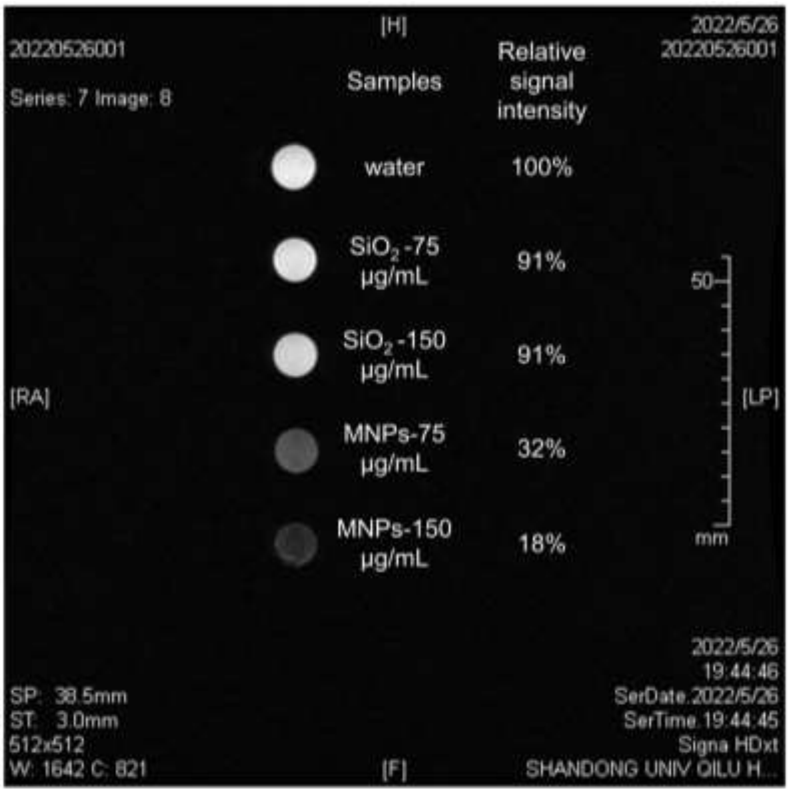

Figure S3. The original data of T_2-weighted MRI image of water, silica of 75 and 150 µg/mL and MNPs of 75 and 150 µg/mL.

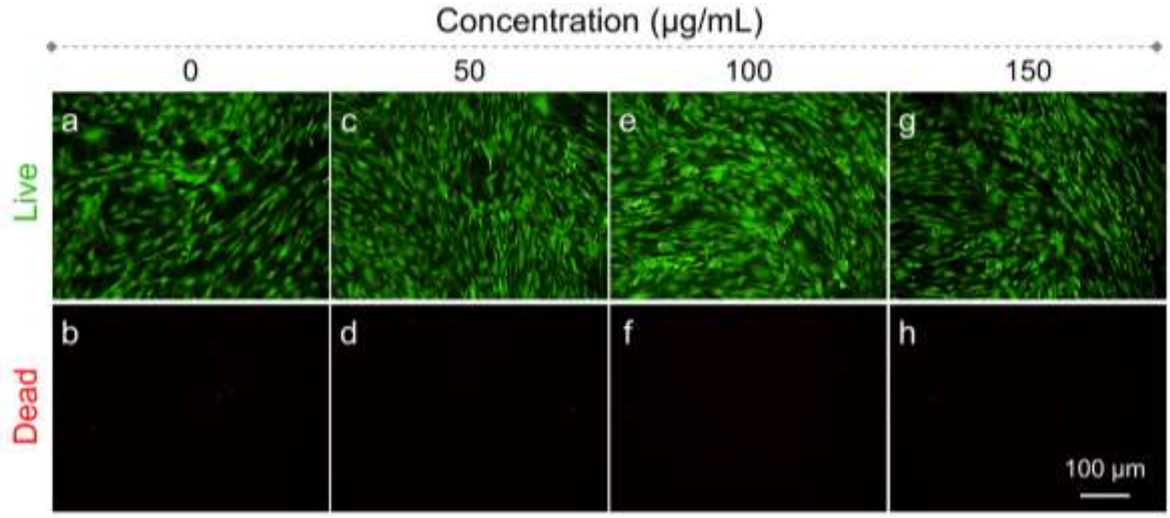

Figure S4. Live/dead cellular staining images after hADSCs were cultured with different concentrations of MNPs for 3 days. Live cells were stained in green and dead cells were in red.

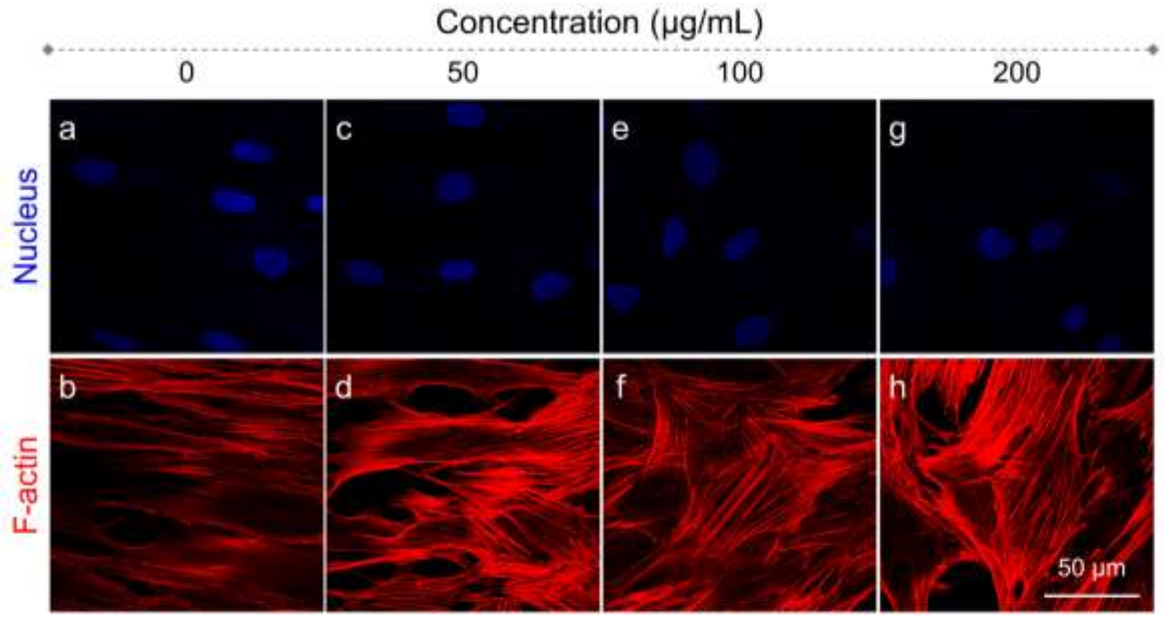

Figure S5. Fluorescence images of cell cytoskeleton when hADSCs were cultured with different concentrations of MNPs for 3 days. F-actin was stained in red with TRITC phalloidin and the nucleus was stained in blue with DAPI.

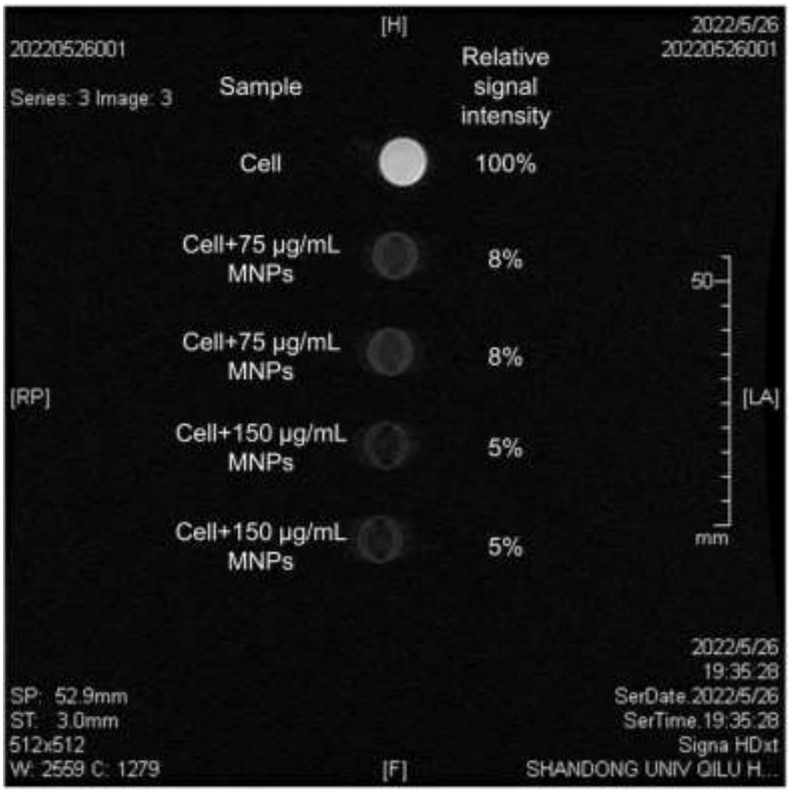

Figure S6. The original data of T_2-weighted MRI images of cell and cell cultured with MNPs of 75 and 150 μg/mL. The relative signal intensity was normalized to that of cell suspension and the counts were 100%, 8%, 8%, 5%, and 5% from top to bottom, respectively.

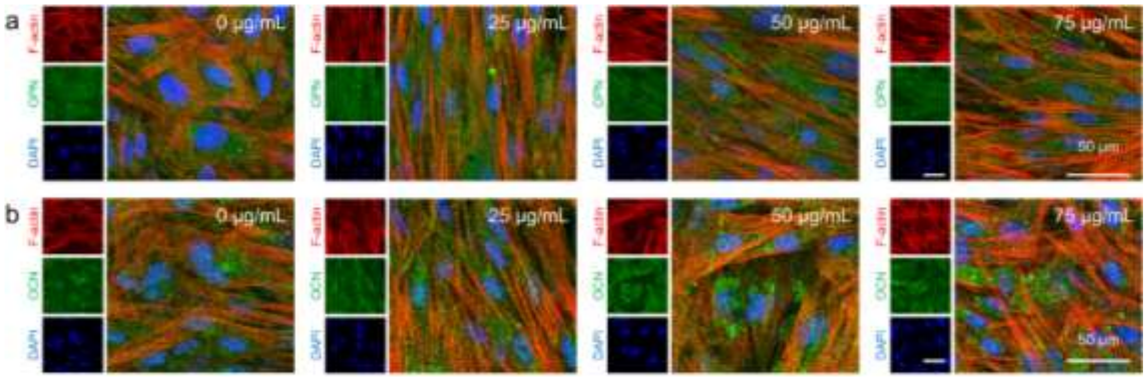

Figure S7. Immunofluorescence staining of OPN and OCN protein after cultured with different concentrations of MNPs 21 days. OPN was stained green, F-actin was stained red and the nucleus was stained blue.

Owing to the long-time differentiation, the green fluorescence of OPN and OCN proteins showed obvious expression among all groups after hADSCs were cultured for 21 days.

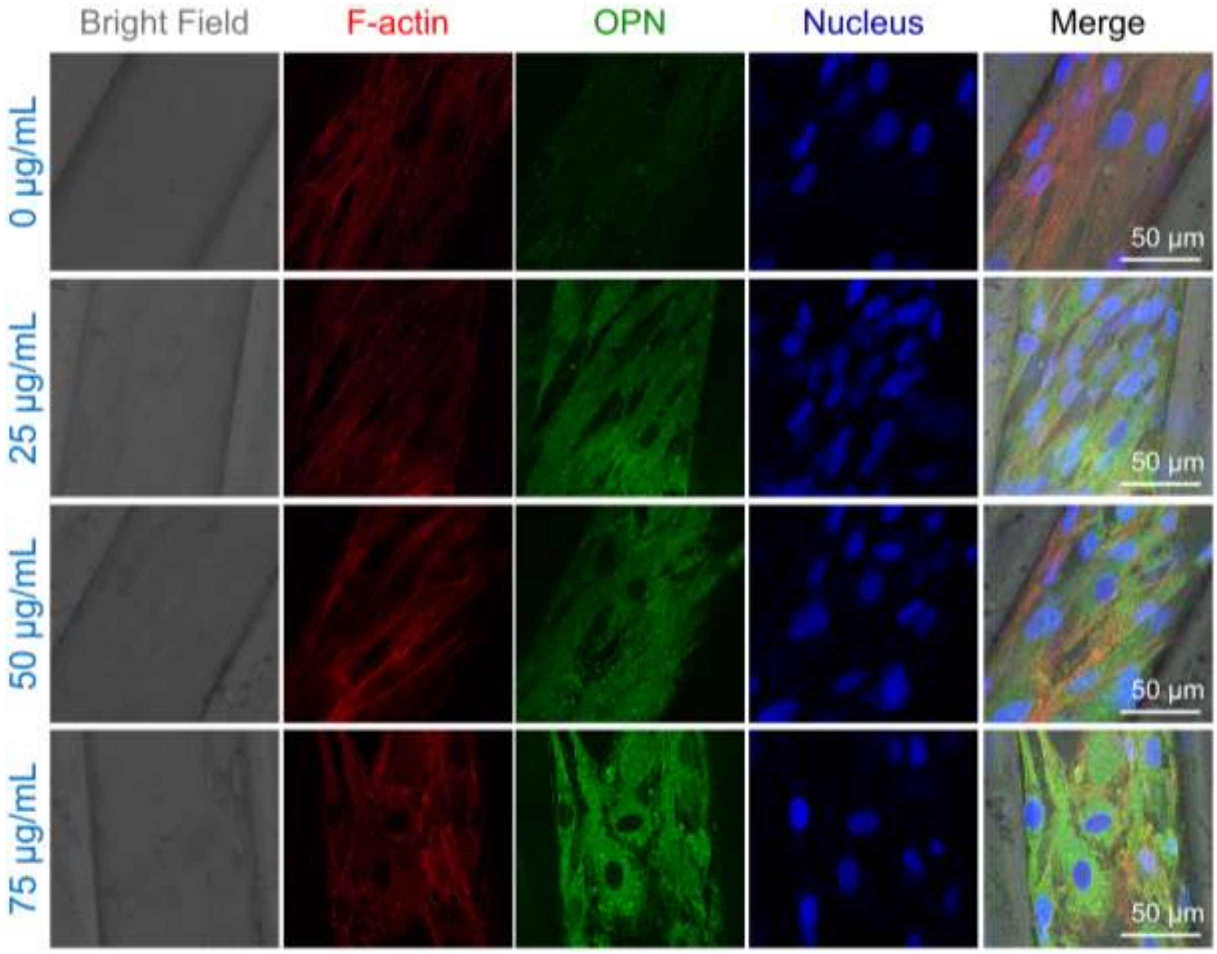

Figure S8. Immunofluorescence staining of OPN protein after cultured with different concentrations of MNPs 7 days on PDMS chips. OPN was stained in green, F-actin was stained in red and the nucleus was stained in blue.

After hADSCs were cultured on the PDMS substrate for 7 days, the expression of osteogenic specific marker OPN was gradually improved and abundant with the increase of MNPs concentration.

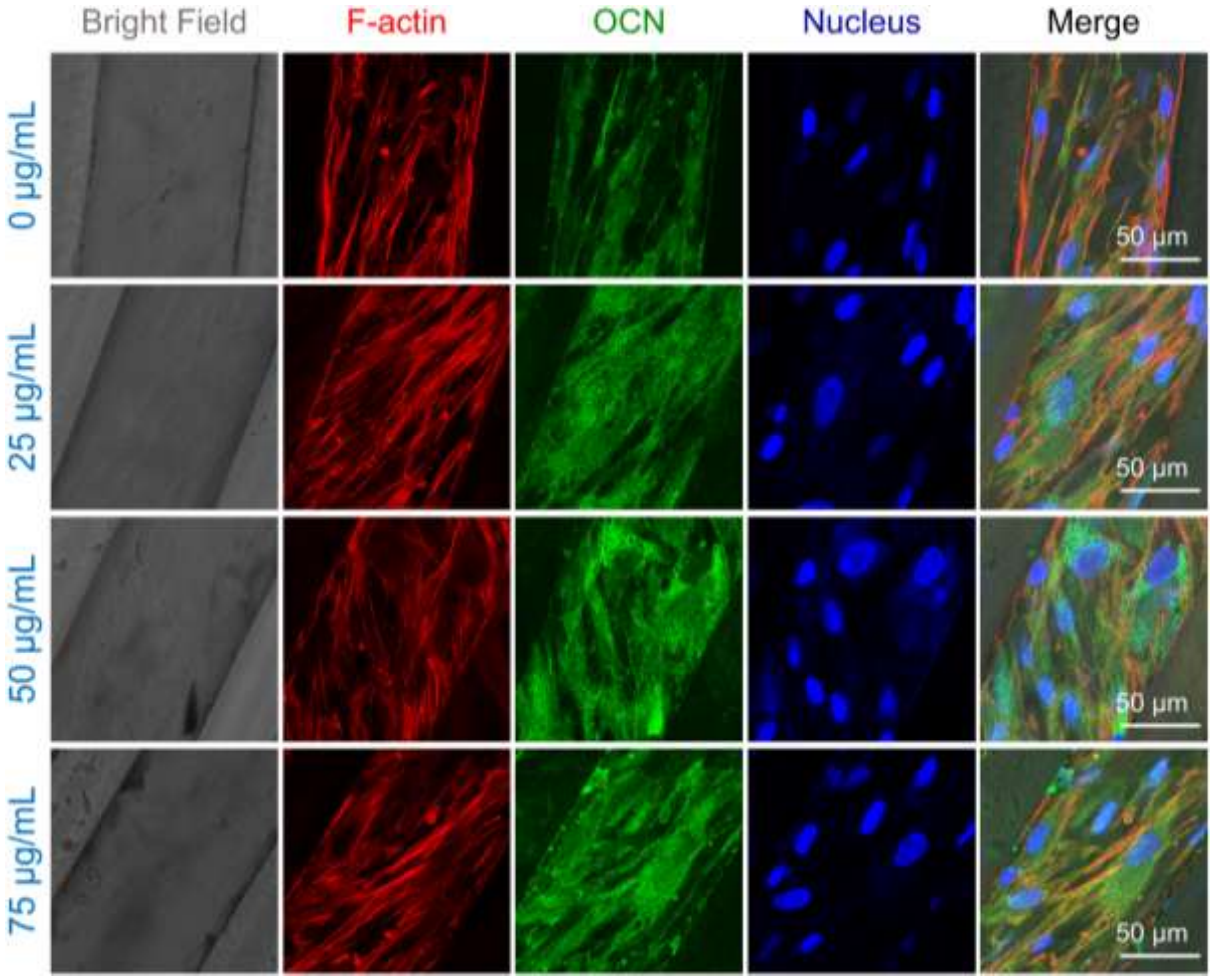

Figure S9. Immunofluorescence staining of OCN protein after cultured with different concentrations of MNPs for 14 days on PDMS chips. OCN was stained in green, F-actin was stained in red and the nucleus was stained in blue.

After hADSCs were cultured on the PDMS substrate for 14 days, the gradual improvement of osteogenic specific marker OCN expression was observed with the increase of MNPs concentration.

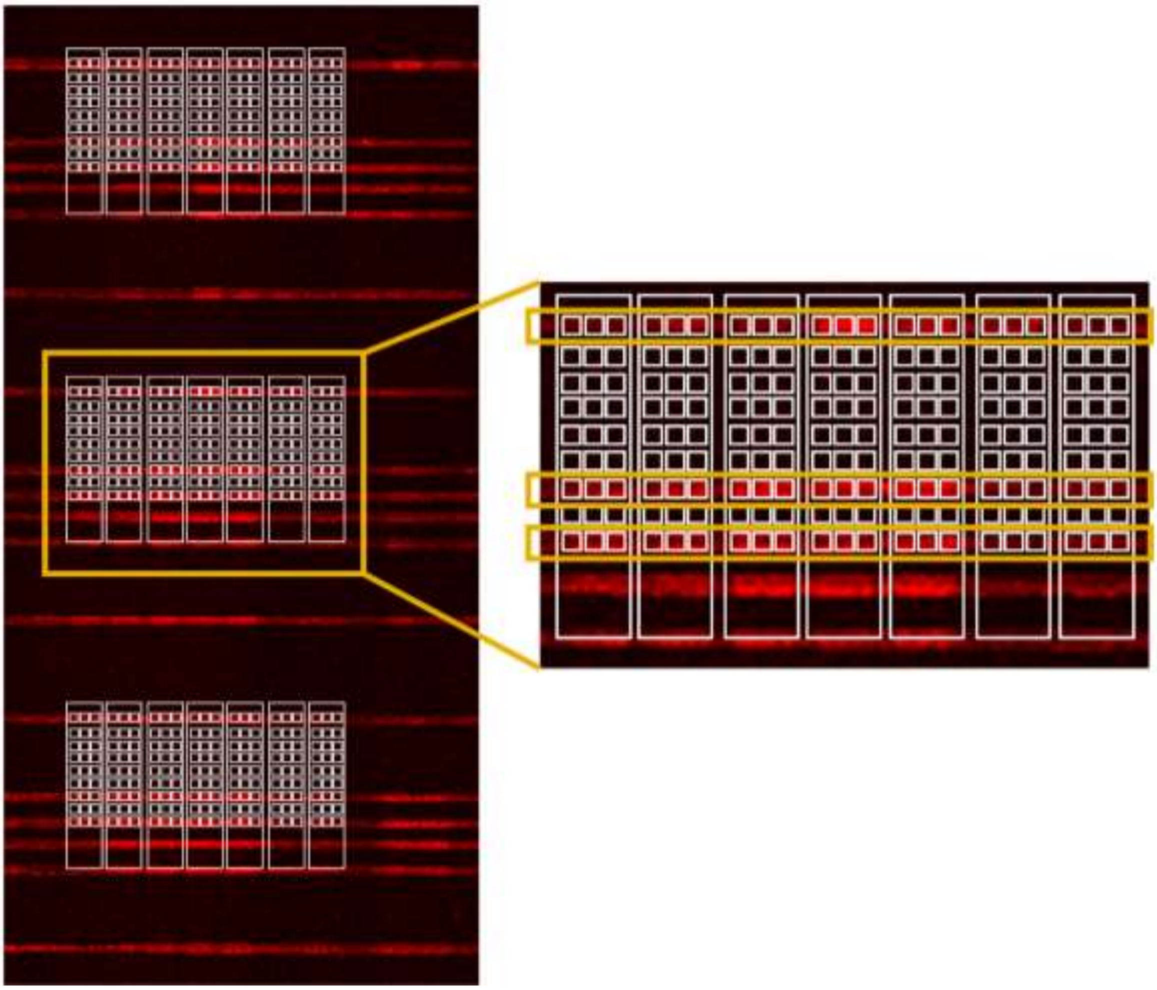

Figure S10. Illustrations for selecting more than 100 points to analyze fluorescence intensity.

After hADSCs were cultured with or without MNPs, microfluidic detection was applied to detect the OCN secretion. Fluorescence images were captured by Genepix 4400A (Molecular Devices) and more than 100 points were randomly selected according to the following way as shown in Figure S6.

In Genepix software, we drew a larger box with the same width as a microchamber, and several smaller boxes (3 × 9) were contained in it to choose pixel points and every smaller box could select four pixels. The fluorescence intensity of every smaller box in one larger box was first exported. Then the fluorescence intensity of one larger box representing a point was counted by averaging the intensity of the 9 smaller boxes in the row (1st, 7th, and 9th) with fluorescent signals, which is the calculation method of the fluorescence intensity of more than

100 points selected randomly mentioned in the article. Then the corresponding statistical analysis was performed by Origin and GraphPad software.

Table S1. Sequences of RT-qPCR primers.

Gene	Forward Primers (5’-3’)	Reverse Primers (5’-3’)
β-actin	CATGTACGTTGCTATCCAGGC	CTCCTTAATGTCACGCACGAT
RUNX2	AATGCCTCCGCTGTTATG	TTCTGTCTGTGCCTTCTTG
OPN	CCTCCTAGGCATCACCTGTG	CCACACTATCACCTCGGCC
OCN	CTGTATCAATGGCTGGGAGC	GCCTGGAGAGGAGCAGAACT
BMP-2	ACCCTTTGTACGTGGACTTC	GTGGAGTTCAGATGATCAGC
